# Feasibility, Safety, Adherence, and Functional Outcomes of a Mobile Application-Facilitated, Unsupervised Remote Cardiac Rehabilitation Program with Exercise Intensity Optimization: A Single-Arm Clinical Trial

**DOI:** 10.3390/jcm15218658

**Published:** 2026-11-06

**Authors:** Kazuhiro Terashima, Ruka Kanazawa, Zhehao Dai, Takaaki Mizuno, Yuji Kono, Takahiro Okumura, Masanobu Yanase, Yohei Otaka, Hideo Izawa

**Affiliations:** 1 Department of Cardiology, Fujita Health University School of Medicine, Toyoake 470-1192, Japantakahiro.okumura@fujita-hu.ac.jp (T.O.); masanobu.yanase@fujita-hu.ac.jp (M.Y.); izawa@fujita-hu.ac.jp (H.I.); 2 Department of Frontier Cardiovascular Science, Graduate School of Medicine, The University of Tokyo, Tokyo 113-0033, Japan; 3 CLINIAL Co., Ltd., Tokyo 151-0053, Japan; takaaki.mizuno@clinial.co.jp; 4 Department of Rehabilitation Medicine, Fujita Health University School of Medicine, Toyoake 470-1192, Japan; yt-kono@fujita-hu.ac.jp (Y.K.); otaka119@mac.com (Y.O.)

**Keywords:** remote cardiac rehabilitation, mobile application, 6-min walk distance, exercise intensity

## Abstract

**Background/Objectives**: Cardiac rehabilitation (CR) is essential for patients with cardiovascular disease (CVD); however, participation rates remain low because of logistical barriers. We developed a mobile application-facilitated, unsupervised remote CR program with exercise intensity optimization and multidisciplinary support. This study aimed to evaluate its feasibility, safety, adherence, and associated changes in functional capacity among patients with CVD. **Methods**: This single-center, single-arm interventional study (jRCTs042230018) enrolled 30 patients with CVD who followed an unsupervised, home-based, mobile application-facilitated CR program for 5 months, with multidisciplinary video counseling every 2 weeks. The primary outcome was the relative change in the 6-min walk distance (6MWD). Secondary outcomes included changes in peak oxygen uptake (VO_2_), ventilatory anaerobic threshold (VAT), ventilation-to-carbon dioxide output slope, and Hospital Anxiety and Depression Scale (HADS) score, as well as program adherence. **Results**: All participants completed the 5-month follow-up and performed a median of 58.5 (interquartile range: 28.0, 77.8) sessions, corresponding to 89.5% (42.8, 118.6) of prescribed sessions (three per week). 6MWD improved from 468.3 ± 98.8 m to 542.6 ± 70.3 m; the mean absolute change was 74.3 m (95% CI, 47.8–100.7; relative change, 18.6%; *p* < 0.001). Peak VO_2_ and VAT increased by 16.7% and 30.1%, respectively. HADS scores decreased modestly. One participant was hospitalized for worsening heart failure (unrelated to the intervention); no other serious cardiovascular events occurred. **Conclusions**: This mobile application-based, unsupervised remote CR program was feasible and associated with improved exercise capacity in patients with CVD. Randomized trials comparing it with center-based CR are warranted.

## 1. Introduction

Cardiovascular disease (CVD) is a leading cause of morbidity and mortality worldwide, imposing substantial burdens on health care systems and individual quality of life. Globally, an estimated 17.9 million people die from CVD annually, accounting for approximately 32% of all deaths and underscoring the need for effective and accessible interventions [1]. In Japan, heart disease is the second leading cause of death and its burden is increasing with population aging [2].

Cardiac rehabilitation (CR) has consistently demonstrated efficacy in improving exercise capacity, reducing hospital readmissions, and improving quality of life in patients with CVD [3]. CR not only lowers recurrence and mortality rates after cardiovascular events but also improves long-term patient outcomes [4,5,6]. Despite these benefits, participation rates in CR programs remain low worldwide, ranging between 10% and 50% in the United States, Canada, and Europe [7,8]. In Japan, participation is even lower, at 4–8% [9]. Conventional CR programs require patients to attend supervised sessions three times per week, often in facilities equipped with specialized exercise machines and staffed by trained personnel. The need to physically attend CR centers contributes directly to low participation, particularly among patients of working age and those living far from these centers [9].

The need for innovative approaches to overcome these barriers has driven the development of remote CR interventions [10]. Although several randomized controlled trials are currently evaluating the effects of remote CR programs on functional capacity in patients with CVD, most existing remote CR models rely on telephone coaching, web-based education, or simple home exercise [11]. Moreover, evidence remains scarce for models that integrate video-guided standing exercise stratified into multiple exercise levels, algorithm-based exercise intensity optimization toward the anaerobic threshold level, and multidisciplinary counseling within a single mobile application. In Japan, where CR participation is particularly low, the development and application of such integrated remote CR models remain especially limited.

To address this gap, we developed a mobile application-facilitated, home-based, unsupervised CR model that combines video-guided exercise sessions stratified into multiple intensity levels, an exercise intensity optimization algorithm driven by patient-entered ratings of perceived exertion, application-based monitoring of daily health data, and remote multidisciplinary counseling every 2 weeks, distinguishing it from general telemedicine or unstructured home exercise. This study aimed to evaluate the feasibility, safety, and adherence of this program in patients with CVD and to describe associated changes in functional capacity.

## 2. Materials and Methods

### 2.1. Study Design and Subjects

This single-arm, single-center interventional study prospectively recruited and screened adult patients aged ≥18 years who were hospitalized with CVD and indicated for CR but were unable or declined to attend standard outpatient CR (see Table S1, which describes the eligibility criteria). Candidates were identified through the usual clinical pathway at our institution: attending physicians or CR staff identified potentially eligible patients during hospitalization or at outpatient visits and referred them to the principal investigator or a sub-investigator, who explained the study, obtained written informed consent, and confirmed eligibility before registration. Eligible patients who could use mobile applications on a smartphone were ultimately included. Patients with clinically unstable cardiovascular conditions, including unstable angina for which treatment had not been completed, uncontrolled arrhythmia, symptomatic severe aortic stenosis, New York Heart Association class IV heart failure, pulmonary embolism, myocarditis or pericarditis, and acute aortic dissection, were excluded. The Certified Review Board of Fujita Health University approved the study protocol (Approval No. CR22-038; 6 February 2023). The study was conducted in accordance with the tenets of the Declaration of Helsinki and complied with local regulations and institutional ethical guidelines. This trial was prospectively registered in the Japan Registry of Clinical Trials (jRCTs042230018) on 1 May 2023, before participant recruitment began.

### 2.2. Consent

Eligible patients meeting the inclusion criteria provided informed consent before study enrollment.

### 2.3. Intervention

The intervention consisted of a 5-month remote CR program facilitated by a mobile application developed by CaTe Inc. (Tokyo, Japan). The 5-month duration was established in accordance with Japan’s universal public health insurance system, under which CR for cardiovascular disease is reimbursed for up to 150 days from the start of treatment [12]. Therefore, we set a 5-month period to maintain comparability. The program comprised four components: (i) home-based exercise training with individualized intensity optimization; (ii) daily self-monitoring of vital signs, body weight, and meals via the application; (iii) multidisciplinary counseling via video call every 2 weeks; and (iv) in-person physician visits. Participants were provided with a smartphone preinstalled with the application. Participants attended in-person visits with their attending physician at baseline and at 1, 3, and 5 months, conducted as part of their regular outpatient visits, at which a medical examination and interview were performed, and additionally received multidisciplinary counseling via video call every 2 weeks, with additional sessions as needed, from a CR team comprising a physician, nurse, and physical therapist. During each session, the team provided guidance on diet, physical activity, and exercise prescription, adjusted the prescribed exercise intensity level according to participant progress, and assessed for signs of worsening heart failure. The team also encouraged continued participation in the program and offered supportive listening when participants raised concerns, including those related to stress; however, no structured psychological intervention was specified in the study protocol. Guidance on medication adherence was provided during both outpatient physician visits and multidisciplinary counseling sessions, although its content and frequency were not systematically recorded for research purposes. Throughout the study, participants continued to receive routine outpatient cardiovascular care; pharmacological therapy was managed by their attending physicians and adjusted as clinically indicated, without a study-specific medication optimization protocol. Concomitant medications, including initiations and discontinuations, were recorded on the case report forms at each visit; dose adjustments were not collected. Cardiovascular medications were defined according to the World Health Organization Anatomical Therapeutic Chemical classification [13] as drugs in anatomical main group C (cardiovascular system) and in groups B01 (antithrombotic agents) and A10BK (sodium–glucose cotransporter 2 inhibitors). Using the mobile application, participants recorded their daily blood pressure, pulse rate, and body weight and photographed each meal. These data were transmitted to the server at the time of entry, exercise records were transmitted at the end of each session, and both were reviewed asynchronously by the CR team on a web-based clinician console. Within the CR team, physicians were primarily responsible for program coordination, review of the data on the clinician console, and setting and adjusting the exercise prescription, and physical therapists performed these tasks under the physicians’ instructions. The application guided participants through three 1-h aerobic exercise sessions per week throughout the 5-month program, including warm-up and cool-down periods, with detailed video instruction tailored to the prescribed exercise intensity. In this study, “unsupervised” refers to the exercise sessions themselves, which participants performed at home without real-time, on-site, or remote supervision by healthcare professionals; participants were monitored remotely through the clinician console and video counseling every 2 weeks. The exercise consisted primarily of standing exercises that did not require large or specialized equipment and could be performed at home following the video guidance. Each exercise session comprised a 10-min warm-up, the main exercise, and a 10-min cool-down. The main exercise consisted of six sets, each with 5 min of exercise followed by 1 min of rest, during which the participant rated their perceived exertion (Borg scale). The exercise prescription, including progression, feedback, and monitoring, is summarized according to the FITT principle (frequency, intensity, time, and type) in Supplementary Table S2.

The intensity level of each exercise menu was determined as described in a previous exercise-load measurement study (internal data), in which 5-min measurements were performed a total of 4792 times for 212 types of exercise in 12 participants. After excluding exercises with large inter-individual variability in intensity level or a large intra-exercise standard deviation (SD) of oxygen uptake (VO_2_), the exercises were classified into eight intensity levels in 0.2-metabolic equivalent of task (MET) increments, with the mean oxygen uptake ranging from 2.4 to 3.8 METs.

Exercise was prescribed at the anaerobic threshold derived from a baseline cycle-ergometer cardiopulmonary exercise test, and the initial intensity level was set from the corresponding MET value (converted from VO_2_). Because peak VO_2_ during treadmill exercise has been reported to be approximately 20% higher than during cycle ergometry in the same individual [14], the initial level set from cycle ergometry tended to be relatively low for standing exercise; the intensity level was therefore increased early. During each rest period, the application evaluated the participant-entered rating of perceived exertion and automatically adjusted the intensity level by one step when it fell outside the target range (Borg 11–13): up when ≤10 and down when ≥14. Heart rate was displayed on an Apple Watch but, as a non-medical device susceptible to motion artifacts, served as a reference only and was not used for intensity adjustment. Post-session summaries and, for selected menus, a camera-based movement-matching score were provided as participant feedback only and did not affect the prescribed intensity (Supplementary Text S1). Consistent with established cardiac rehabilitation guidelines [15], in which exercise is prescribed at the anaerobic threshold level and the general framework for risk management is applied uniformly regardless of the underlying cardiovascular diagnosis, the same eligibility criteria, anaerobic threshold-based exercise intensity optimization protocol, and safety monitoring procedures were applied across all diagnostic groups. The individualized, anaerobic threshold-based prescription accounted for differences in baseline exercise capacity among participants. Given the sample size, no diagnosis-specific subgroup analyses were planned and as this was a single-arm study, a comparison group was not included.

### 2.4. Outcome Measurements

The primary outcome was the relative change in the 6-min walk distance (6MWD) after the 5-month program compared with baseline. Secondary outcomes included adherence to the prescribed exercise sessions; changes from baseline to 5 months in cardiopulmonary exercise test performance, including peak VO_2_, ventilatory anaerobic threshold (VAT), and ventilation-to-carbon dioxide output slope; and the change in perceived mental health symptoms assessed using the Japanese version of the Hospital Anxiety and Depression Scale (HADS) [16]. Adherence was defined as the number of aerobic exercise sessions automatically logged by the application relative to the prescribed three sessions per week, summarized as the total number of recorded sessions, the percentage of prescribed sessions completed, and the proportion of participants meeting weekly thresholds (≥1, ≥2, and ≥3 sessions/week). No threshold defining adequate adherence was set a priori. Sessions without an application record were treated as not performed. Feasibility was described using four domains: recruitment (the proportion of patients assessed for eligibility who were enrolled), retention (the proportion of participants who completed the 5-month follow-up), adherence to the prescribed exercise sessions, and safety (adverse events leading to discontinuation of the program). Adherence to other program components (daily health-data uploads and counseling sessions) was not formally quantified (see Supplementary Text S1 for additional details on the intervention platform and adherence measurement). Safety outcomes included prespecified adverse events occurring during the study period, including newly diagnosed myocardial infarction, stable or unstable angina, aortic dissection, and ischemic or hemorrhagic stroke, as well as adverse events leading to discontinuation of the program. As specified in the protocol, all adverse events, regardless of their causal relationship with the program, and concomitant medications were assessed throughout the study period. For each event, the investigator recorded the event name, onset date, severity (mild, not interfering with daily life; moderate, interfering with daily life to some extent; or severe, preventing daily life), and causal relationship to the program (related or unrelated), which was judged by considering the pattern and timing of onset, concomitant medications, comorbidities, and medical history. Before each exercise session, participants completed a 15-item pre-exercise self-check list in the application based on the Physical Activity Reference for Health Promotion 2013 issued by the Japanese Ministry of Health, Labour and Welfare [17] (e.g., chest pain, shortness of breath or palpitations with slight exertion, fever, and headache or dizziness); if any item was selected, the application instructed the participant to stop rehabilitation and did not allow the session to start. At enrollment, participants were instructed in writing to contact their attending physician immediately if they experienced any unusual symptoms or change in their condition, and any necessary care was provided as part of usual care. In addition, the CR team reviewed the uploaded health data and held remote counseling every 2 weeks; any concern was discussed with the attending physician, who decided whether to suspend exercise or arrange an outpatient visit. No real-time monitoring was performed during home exercise.

The 6-min walk test was performed along a 30-m straight corridor by trained physical therapists or other qualified staff in accordance with the American Thoracic Society statement [18], with standardized encouragement provided at 1-min intervals. Cardiopulmonary exercise testing was performed on an electronically braked cycle ergometer using a symptom-limited, individualized ramp protocol (10–15 W/min according to estimated exercise capacity), with breath-by-breath gas analysis (Minato Medical Science, Osaka, Japan). All tests were supervised by a physician, who also performed the analysis. Peak VO_2_ was defined as the highest VO_2_ achieved during the test, and a respiratory exchange ratio of >1.05 was required to confirm adequate effort. VAT was determined by the V-slope method [19], supplemented by the ventilatory equivalent method (trends in VE/VO_2_ and VE/VCO_2_, where VE denotes minute ventilation); when the two methods disagreed, the lower value was adopted. The HADS consists of two seven-item subscales (anxiety and depression), each scored 0–21, with higher scores indicating more severe symptoms [16]. Participants completed the HADS as a self-administered questionnaire during clinic visits at baseline and 5 months.

### 2.5. Statistical Analysis

The sample size was calculated, as specified in the protocol, based on the primary outcome, defined as the relative change in 6MWD from baseline to 5 months. In a previous rehabilitation trial, the mean relative change in 6MWD in the intervention group was approximately 9%, with a wide SD of 23% [20]. In view of this wide variability, a conservative threshold of 5%, between no change and the mean change in that trial, was set as the minimum improvement to be demonstrated, whereas an improvement of ≥15% was considered clinically promising. Assuming an expected increase of 15%, an SD of 30%, a two-sided alpha level of 0.05, and a power of 80%, 73 participants were required to detect this difference against the 5% threshold using a one-sample *t*-test [21], and the target sample size was set at 100. Because enrollment proceeded more slowly than planned (30 participants between July 2023 and March 2024), the planned 100 participants could not have been enrolled within the study period. In view of this recruitment status and the results of the interim analysis, the investigators closed enrollment at 30 participants, as reported in the summary of results in the trial registry (jRCTs042230018). Although percentage change from baseline has lower statistical power than absolute change or analysis of covariance, the relative change in 6MWD was prespecified as the primary outcome to enable direct comparison with prior CR cohorts reporting relative improvements; the sample-size estimate incorporated a conservatively inflated SD to mitigate this inefficiency [22].

Outcome analyses were performed in the full analysis set, which included all enrolled participants except those determined to be ineligible after enrollment or who did not participate in the program. The primary analysis evaluated the relative change in 6MWD from baseline to 5 months using a one-sample *t*-test with μ = 5% and a two-sided α of 0.05. Results are presented as the sample mean with 95% CI. Absolute changes from baseline to 5 months were additionally analyzed using paired *t*-tests. Relative changes for the primary and secondary outcomes were calculated as the individual percentage change from baseline for each participant and are reported as the mean percentage change with 95% CI. Where results are compared with those of previous cohorts reporting group-level percentages, the corresponding group-level relative change is additionally provided for descriptive comparison only.

Adherence was summarized descriptively as the median (interquartile range [IQR]) number of application-recorded exercise sessions, the median (IQR) percentage of prescribed sessions completed, and the proportion of participants meeting weekly exercise-frequency thresholds, without formal statistical testing. Secondary continuous outcomes were analyzed as absolute mean changes with 95% CIs and paired *t*-test comparisons; for peak VO_2_ and VAT, relative changes from baseline were additionally reported. Secondary outcome analyses were exploratory, as prespecified in the protocol. As a post hoc sensitivity analysis, *p*-values for the five secondary efficacy outcomes (changes in peak VO_2_, VAT, ventilation-to-carbon dioxide output slope, and HADS-anxiety and HADS-depression scores) were adjusted for multiplicity using the Holm method.

All 30 participants completed the baseline and 5-month assessments for the 6-min walk test and cardiopulmonary exercise testing; therefore, no imputation was required for these outcomes. HADS data at 5 months were unavailable for three participants, who were excluded from the HADS analysis (*n* = 27). Among the remaining questionnaires, three single missing-item responses, all on the depression subscale, were assigned a score of 0 (the least symptomatic response) before subscale totals were calculated. Because this approach may favor improvement, a post hoc sensitivity analysis assigning a score of 3 (the most symptomatic response) was also performed. Health-data uploads and counseling attendance were not analyzed as outcomes; therefore, no missing-data procedures were applied to these program components. Safety analyses included all patients who completed at least one session of the program.

All statistical analyses were performed using R (R Foundation for Statistical Computing, Vienna, Austria), version 4.0.3 for the original analyses and version 4.4.1 for the analyses added during revision.

## 3. Results

### 3.1. Participant Characteristics

Between July 2023 and March 2024, 48 patients were screened for eligibility, of whom 30 (23 male [77%]; mean age, 61.6 years) were enrolled and received the mobile application-facilitated remote CR intervention. Detailed reasons for exclusion are listed in Figure S1. Thus, 30 of the 48 patients assessed for eligibility (62.5%) were enrolled, and all 30 participants completed the 5-month follow-up (retention, 100%). No adverse event led to discontinuation of the program. All 30 participants had a respiratory exchange ratio of >1.05 on the baseline cardiopulmonary exercise test. The indications for CR—that is, the index CVD for inclusion—were mainly myocardial infarction (19 [63%] patients), followed by chronic heart failure (7 [23%]), angina pectoris (3 [10%]), and history of cardiac surgery (1 [3%]). Other baseline characteristics are summarized in Table 1. The mean left ventricular ejection fraction was 48% ± 11%, and the median N-terminal pro–B-type natriuretic peptide was 651 (IQR 213, 1147) pg/mL, indicating mildly reduced cardiac function with only mild-to-moderate natriuretic peptide elevation. Common comorbidities included dyslipidemia (83%), hypertension (63%), and diabetes mellitus (37%), and baseline functional capacity was moderately reduced. Cardiovascular medications at enrollment commonly included β-blockers (90%), renin–angiotensin system inhibitors (87%), statins (83%), and antiplatelet agents (83%) (Table 1). During the intervention period, changes in cardiovascular medications (initiation or discontinuation) were recorded in 11 of the 30 participants (37%). According to the medical records, no participant received any other outpatient or home-based rehabilitation program during the intervention period; one participant received inpatient rehabilitation during a hospitalization for worsening heart failure (Section 3.5).

### 3.2. Program Adherence

All 30 participants completed the 5-month follow-up. The median number of exercise sessions recorded through the mobile application during the study period was 58.5 (IQR 28.0, 77.8), corresponding to a median of 89.5% (IQR 42.8, 118.6) of the prescribed sessions. Overall, 24 (80%) participants exercised at least once weekly, 18 (60%) at least twice weekly, and 11 (37%) at least three times weekly. The percentage of the prescribed sessions (three sessions per week) completed by each participant ranged from 12.4% to 201.1%: 11 participants (37%) completed ≥100% of the prescribed sessions, 5 (17%) completed 80% to <100%, 4 (13%) completed 50% to <80%, and 10 (33%) completed <50% (Figure S2).

### 3.3. Functional and Cardiopulmonary Outcomes

**Figure 1:**
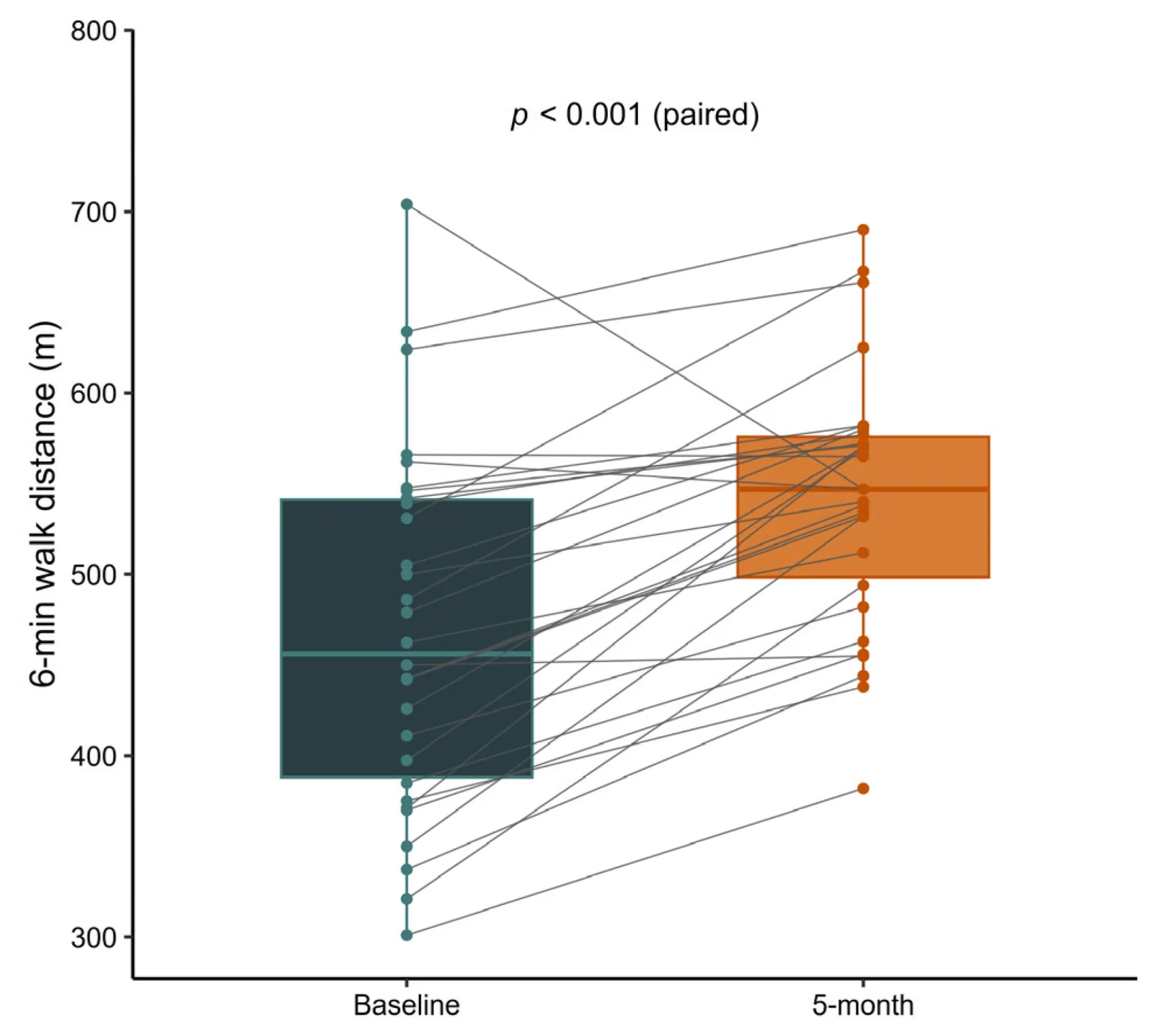
Paired comparison of the 6-min walk distance between baseline and 5 months.

Functional capacity, as assessed by the 6MWD, improved from a mean ± SD of 468.3 ± 98.8 m at baseline to 542.6 ± 70.3 m at 5 months; the mean absolute change was 74.3 m (95% CI, 47.8–100.7 m; Figure 1; paired *t*-test, *p* < 0.001). The mean relative change in 6MWD was 18.6% (95% CI, 12.1–25.1%; one-sample *t*-test, *p* < 0.001 for μ = 5%).

**Figure 2:**
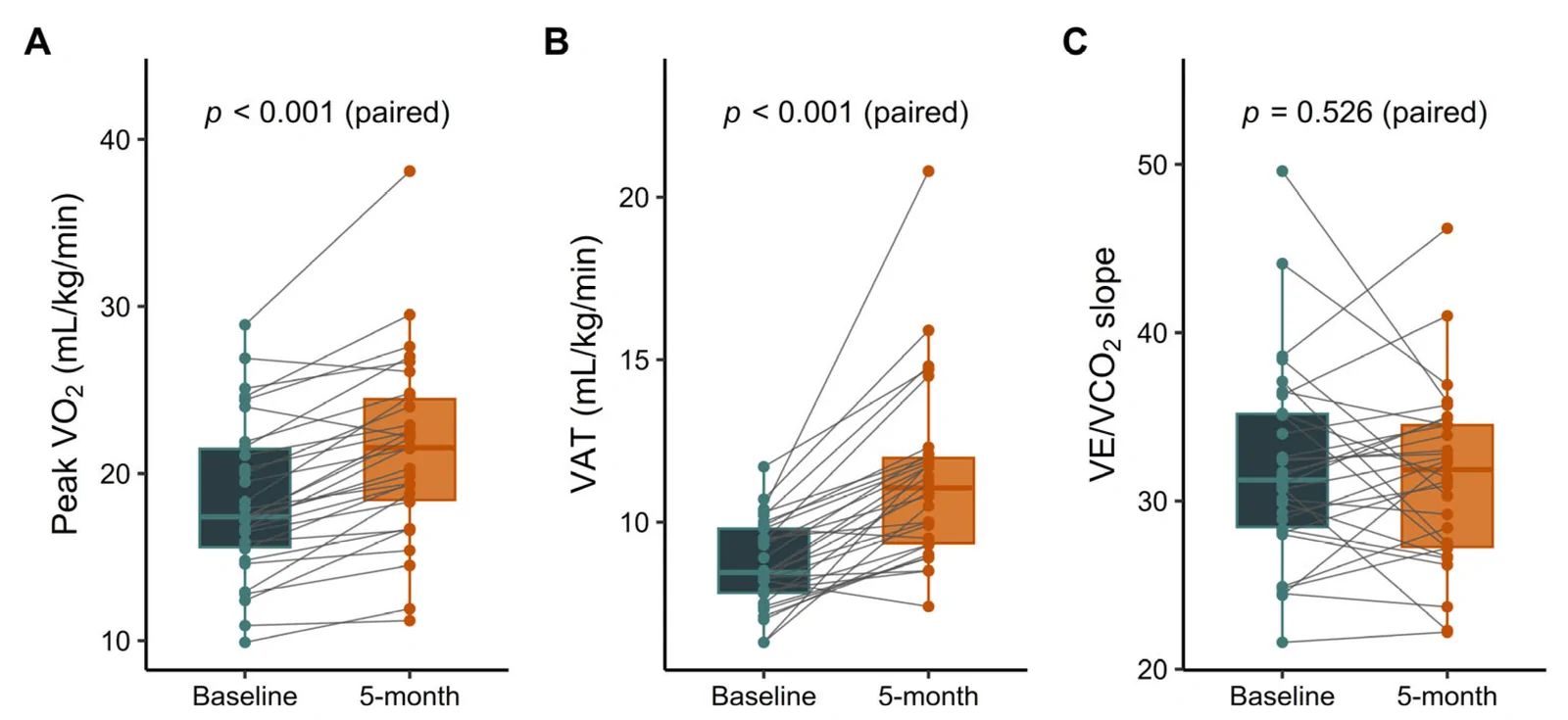
Paired comparisons of peak oxygen uptake (**A**), ventilatory anaerobic threshold (**B**), and the ventilation-to-carbon dioxide output slope (**C**) measured by cardiopulmonary exercise testing between baseline and 5 months. Abbreviations: VAT, ventilatory anaerobic threshold; VE/VCO_2_, ventilation-to-carbon dioxide output; VO_2_, oxygen uptake.

The mean peak VO_2_ increased from 18.4 ± 4.7 mL/kg/min at baseline to 21.4 ± 5.5 mL/kg/min at 5 months, with a mean absolute change of 3.0 mL/kg/min (95% CI, 2.1–3.8 mL/kg/min; Figure 2A; paired *t*-test, *p* < 0.001), corresponding to a mean relative change of 16.7% (95% CI, 12.2–21.2%). The mean VAT increased from 8.7 ± 1.3 mL/kg/min to 11.3 ± 2.7 mL/kg/min; the mean absolute change was 2.6 mL/kg/min (95% CI, 1.8–3.4 mL/kg/min; Figure 2B; paired *t*-test, *p* < 0.001) and the mean relative increase was 30.1% (95% CI, 21.0–39.1%). By contrast, the mean ventilation-to-carbon dioxide output slope did not change significantly, from 32.0 ± 6.0 to 31.4 ± 5.3 (paired *t*-test, *p* = 0.526; Figure 2C), with a mean absolute change of −0.61 (95% CI, −2.55 to 1.33).

### 3.4. Mental Health Outcomes

Among the 27 participants with HADS data at both time points, the mean HADS-anxiety score decreased from 3.3 ± 2.7 to 2.4 ± 2.2 (paired *t*-test, *p* = 0.034), and the mean HADS-depression score decreased from 5.7 ± 4.3 to 3.8 ± 3.1 (paired *t*-test, *p* = 0.013). In the sensitivity analysis assigning the most symptomatic response to missing items, the HADS-depression score decreased from 5.8 ± 4.4 to 4.0 ± 3.3 (*p* = 0.017). After the post hoc Holm adjustment for the five secondary efficacy outcomes, the changes in peak VO_2_ (adjusted *p* < 0.001), VAT (adjusted *p* < 0.001), and HADS-depression score (adjusted *p* = 0.040) remained statistically significant, whereas the change in HADS-anxiety score did not (adjusted *p* = 0.068); the adjusted *p*-value for the ventilation-to-carbon dioxide output slope was 0.526.

### 3.5. Safety Outcomes

Regarding safety, no cases of newly developed myocardial infarction, stable or unstable angina, aortic dissection, ischemic stroke, or hemorrhagic stroke occurred during the study period. During the intervention period, 37 adverse events occurred in 18 participants (60%); 35 were mild and 2 were moderate; none were severe, and none were judged to be related to the program or led to discontinuation (Table S3). One participant, whose index condition was chronic heart failure, was hospitalized for worsening heart failure of moderate severity; because hospitalization was required, this event was classified as a serious adverse event. The participant developed exertional dyspnea and pleural effusion approximately 7 weeks after starting the program. The worsening was identified by the CR team during remote counseling before hospitalization; after consultation with the attending physician, exercise was temporarily suspended and the participant was referred for an outpatient visit, at which diuretics were adjusted. The participant was subsequently hospitalized, partly because of anxiety about the worsening symptoms and social circumstances. Because the deterioration had been identified and exercise had been suspended before hospitalization, the attending physician judged the event to reflect the course of the underlying chronic heart failure and to be unrelated to the program, after considering the pattern and timing of onset, concomitant medications, comorbidities, and medical history. The participant received inpatient rehabilitation during the hospitalization; after discharge, the exercise prescription was modified, and the participant resumed the remote CR program and completed the 5-month follow-up. No other serious adverse events occurred.

## 4. Discussion

In this single-arm study, participation in a multicomponent, home-based, unsupervised remote CR program supported by a mobile application was associated with increased functional capacity; all participants completed the 5-month follow-up, and no adverse event was judged to be related to the intervention. One of the most notable observations is the exercise frequency achieved by the participants. Conventional outpatient CR programs generally involve supervised exercise sessions three times weekly; however, adherence to prescribed exercise is often suboptimal [7,8,9]. An analysis of the nationwide Japanese health insurance claims database revealed remarkably low exercise frequency among patients who underwent outpatient CR after acute coronary syndrome, with a median of only nine exercise sessions (IQR 3, 17) within 180 days after hospitalization and only 3.1% of patients exercising at least twice weekly [23]. Although no adherence threshold was set a priori and these data are not directly comparable because that cohort received facility-based outpatient CR, the substantially greater engagement in the present study—a median of 58.5 (IQR 28.0, 77.8) sessions and 60% of participants exercising at least twice weekly—may have contributed to the observed improvements in functional capacity. Internationally, a meta-analysis of 14 studies reported that patients enrolled in CR attended a mean of 66.5% (range across studies, 36.7–84.6%) of prescribed sessions [24], and only 26.9% of Medicare beneficiaries in the United States who participated in CR completed at least 36 sessions [25]. Although adherence has been defined differently across studies, the median of 89.5% of prescribed sessions completed in the present study, with 37% of participants completing all prescribed sessions, compares favorably with these reports.

The high exercise frequency observed in the present study may partly reflect the unsupervised and flexible nature of the remote CR program, a phenomenon also reported in physical activity interventions for other diseases [26]. Although supervised CR programs provide real-time monitoring and direct feedback, they may also impose logistical burdens that limit adherence, flexibility, and patient autonomy, potentially contributing to lower adherence. By contrast, the mobile application-facilitated remote program empowered participants to exercise independently at their convenience while still receiving regular monitoring and feedback through application-based tracking and video-based counseling. The ability to incorporate exercise into daily routines without attending facility-based exercise sessions may have contributed to sustained engagement over the 5-month study period [7,8].

Our findings should be interpreted in light of the randomized evidence on remote care and remote CR. Randomized trials of telemonitoring-based remote management in heart failure, which did not include structured exercise training, have yielded inconsistent results [27,28,29]: TIM-HF2 reduced the percentage of days lost due to unplanned cardiovascular hospitalization or all-cause death, whereas TEN-HMS and BEAT-HF did not meet their primary endpoints. Meta-analyses of randomized trials have reported improvements in exercise capacity with cardiac telerehabilitation that were similar to, or slightly greater than, those with conventional or center-based programs, although the pooled effects were small and the included trials were small [11,30]. The 2026 European Society of Cardiology (ESC) Guidelines state that cardiac telerehabilitation or hybrid telerehabilitation should be considered as an alternative to center-based CR in patients with coronary syndromes or heart failure (Class IIa). The same guidelines note that comparisons are limited by small sample sizes, follow-up of up to 12 months, and heterogeneous interventions and comparators, and that adverse events were recorded in only 43% of the trials in one review [11,31]. In a network meta-analysis of 139 randomized trials in heart failure, mean differences versus usual care ranged from 1.76 to 3.10 mL/kg/min for peak VO_2_ across delivery modes and from 36.8 to 84.8 m for 6MWD across home-based, center-based, and hybrid delivery [32]. In a randomized trial of patients aged ≥65 years who had declined center-based CR, a 6-month home-based, mobile-guided CR program increased peak VO_2_ by 1.6 mL/kg/min, a between-group difference of 1.2 mL/kg/min compared with no CR [33]. The within-participant changes in the present study (3.0 mL/kg/min in peak VO_2_ and 74.3 m in 6MWD) were numerically larger than these between-group differences. However, uncontrolled within-group changes also include natural recovery after the index event, regression to the mean, and learning effects, so they cannot be compared directly with randomized between-group estimates and are likely to overestimate the effect attributable to the program. Previous studies of remote CR using a mobile application or remote biological-signal monitoring were also small single-arm or pilot studies [34,35]. Accordingly, the present data cannot establish whether this program is non-inferior to or superior to center-based CR, or whether it increases CR participation. To address these questions, a multicenter, randomized non-inferiority trial comparing an updated version of this program with conventional outpatient CR in patients with heart failure is currently underway (jRCT2042260056).

Notably, improvements in 6MWD, peak VO_2_, and VAT were observed in a program whose exercise component consisted of equipment-free standing exercises. The absolute gains—3.0 mL/kg/min in peak VO_2_ (16.0% relative to the baseline group mean), 74.3 m in 6MWD (15.9%), and 2.6 mL/kg/min in VAT (29.7%)—are numerically within the range reported for supervised center-based CR using cycle ergometers or treadmills, including peak VO_2_ improvements of 1.9–3.5 mL/kg/min (~12–16%) [36,37]. Regarding clinical relevance, the mean improvements in 6MWD (74.3 m; 95% CI, 47.8–100.7 m) and peak VO_2_ (3.0 mL/kg/min; 95% CI, 2.1–3.8 mL/kg/min), including the lower bounds of their 95% CIs, exceeded the minimal clinically important differences (MCIDs) of 30 m and 1 mL/kg/min referred to in the 2026 ESC Guidelines [31], and 25 of 30 participants (83%) reached or exceeded the threshold for each outcome. The improvement in 6MWD also exceeded the MCID of 25 m reported for patients with coronary artery disease after acute coronary syndrome [38]. No widely accepted MCID has been established for VAT; therefore, the change in VAT should be interpreted cautiously. In the absence of a control group, the extent to which these improvements are attributable to the program cannot be determined. Because no large exercise equipment is required, the program may require less equipment and space than center-based CR. However, the costs of the application, the devices lent to participants, and staff time for remote monitoring and counseling were not assessed, and neither cost-effectiveness nor scalability was evaluated in the present study. A systematic review of randomized controlled trials reported that cardiac telerehabilitation could lead to similar or lower long-term costs than center-based CR [39]; whether this applies to the present program remains to be determined.

The recently published 2026 ESC Guidelines on cardiac rehabilitation emphasize that CR is a comprehensive, multidisciplinary intervention that should not be equated with exercise training alone; in addition to exercise training, its core components include patient assessment, self-management and education, optimization of cardiovascular pharmacotherapy, physical activity and nutritional counseling, and psychosocial management, and telerehabilitation and hybrid models are recognized as delivery options alongside center-based CR [31]. In the present program, unsupervised home-based exercise training with individualized intensity optimization was embedded within a remotely supported, multicomponent framework that included patient education on diet, physical activity, and medication adherence; daily self-monitoring; and ongoing assessment of clinical stability through in-person physician visits and multidisciplinary counseling every 2 weeks, while pharmacological therapy was managed through routine outpatient care. Basic psychosocial support, including encouragement and supportive listening, was provided during multidisciplinary counseling; however, no structured, protocol-defined psychological or social-support intervention was included, and pharmacotherapy optimization was not protocolized within the program. Although psychological symptoms and safety outcomes were also assessed, the primary outcome and adherence measurement focused on exercise-related endpoints, and the contributions of individual non-exercise components could not be determined separately. The present findings should therefore be interpreted as supporting the feasibility of this remotely supported delivery model, rather than as an evaluation of comprehensive CR in its entirety.

Regarding safety, systematic reviews of home-based and remotely delivered CR have reported a very low incidence of exercise-related severe or serious adverse events, estimated at approximately 1 per 23,823 and 1 per 53,770 patient-hours of exercise, respectively, with no exercise-related deaths [40,41]. In the present study, no exercise-related serious adverse event occurred during 1679 application-recorded sessions; however, this exposure is too limited to establish safety. Safety in this program relied on pre-participation screening including cardiopulmonary exercise testing, automatic intensity regulation based on the rating of perceived exertion, and asynchronous remote monitoring, whereas no real-time emergency-response system was established; these elements will be important considerations for broader implementation of mobile health (mHealth)-based CR.

This study was limited by its single-center, single-arm design with a small sample, smaller than originally planned, and no control group or blinding; the observed changes, therefore, cannot be attributed causally to the intervention and may reflect natural recovery (particularly after myocardial infarction, the most common index diagnosis), regression to the mean, or a learning effect on the 6MWD. Nevertheless, several findings support the robustness of the observed improvement. Although the sample was smaller than originally planned, the lower bound of the 95% CI for the mean relative change in 6MWD (12.1%) exceeded the prespecified 5% threshold, and the improvement was consistent between two different measures of functional capacity, the 6MWD and peak VO_2_, with 25 of 30 participants (83%) reaching or exceeding the MCID for each outcome. We therefore consider the improvement in functional capacity observed in this cohort to be robust, although controlled trials are required to determine the extent to which it is attributable to the program. A further limitation is the clinical heterogeneity of the cohort: the index diagnoses included myocardial infarction (63%), chronic heart failure (23%), angina pectoris (10%), and history of cardiac surgery (3%), conditions that differ in pathophysiology, expected trajectory of recovery, and responsiveness to exercise training. Although a uniform anaerobic threshold-based prescription was applied across diagnoses in accordance with current guidelines, the sample size precluded diagnosis-specific analyses, and the interval from the index hospitalization to enrollment was not systematically recorded. Larger studies with diagnosis-stratified designs are needed to determine whether the observed changes are consistent across cardiovascular conditions. In addition, no participant was enrolled with an arrhythmia as the index condition. Home-based and digitally supported CR has been proposed to improve access for patients with atrial fibrillation [42], and the 2026 ESC Guidelines state that telerehabilitation or hybrid CR should be considered in this population [31]; however, because of disease-specific concerns such as exercise-triggered recurrence of atrial fibrillation [42], the safety and effectiveness of the present unsupervised program in these patients require separate evaluation. Enrollment was restricted to patients able and willing to use a smartphone application; long-term follow-up was not performed; adherence was quantified only for the exercise component and summarized cumulatively without analysis of temporal trends; application usability was not assessed with a validated instrument; and no system was established for on-site response to acute events during unsupervised home exercise—a central safety consideration for remote CR. Moreover, the small sample size was insufficient to establish the safety of the program, and adverse-event relatedness was assessed by the attending physician, who also conducted the study visits, rather than by an independent adjudication committee. In addition, while basic psychosocial support was provided during counseling, no structured, protocol-defined psychological or social-support intervention was included, and pharmacological therapy was managed by attending physicians as part of routine care without protocolized optimization; although initiations and discontinuations of concomitant medications were recorded, dose adjustments were not systematically collected in the case report forms, and the influence of medication changes on the outcomes was not analyzed. Therefore, the observed changes cannot be attributed to the exercise intervention alone, and the contributions of concomitant pharmacological management and other non-exercise components could not be evaluated separately. Furthermore, the content and frequency of counseling were not systematically recorded for research purposes, precluding assessment of the fidelity of these components and of the contribution of psychosocial support to the observed changes in HADS scores. Finally, because this study was funded by CaTe Inc., the developer of the evaluated program, and several authors are affiliated with the company (see Conflicts of Interest), the potential for sponsor-related bias should be considered when interpreting the results. To minimize this risk, the protocol, including the prespecified primary endpoint, was approved by the Certified Review Board, which also reviewed the conflict-of-interest management plan under the Clinical Trials Act, and the study was registered before enrollment. Outcome measurements, including the 6-min walk test and cardiopulmonary exercise testing, were performed by clinical staff at Fujita Health University Hospital. Data management and monitoring, including verification of case report form data against medical records, were performed by an external contract research organization, and an external audit was conducted. The statistical analysis for the study report was requested from the designated analysis manager at Fujita Health University after database lock. Supplementary Text S2 describes the study limitations in full.

The strengths of this study include the prospective evaluation of a remotely supported CR program incorporating unsupervised, equipment-free home exercise in patients who had declined or were unable to attend outpatient CR—a population rarely captured in CR trials—with automatically and objectively recorded exercise sessions over 5 months and cardiopulmonary exercise testing before and after the program. Practically, these findings suggest that improvements in functional and cardiopulmonary capacity may be attainable with home-based exercise that requires no specialized equipment; if confirmed in controlled trials, this could inform program design where center capacity or travel distance limits participation. Future work should confirm these findings in multicenter randomized trials, incorporate medical-grade heart rate monitoring and real-time emergency-response pathways, refine prescription algorithms for the MET difference between exercise modalities, capture component-level engagement to identify which elements drive benefit, integrate structured psychosocial support and protocolized pharmacotherapy optimization within the program, and include formal economic evaluations, such as cost-effectiveness analyses.

## 5. Conclusions

Participation in this mobile application-facilitated remote CR program was associated with high exercise frequency and within-participant improvements in functional capacity in patients with CVD. The high exercise frequency may reflect the flexibility of unsupervised participation; however, the single-arm design precludes causal inference. Whether this program can expand access to CR and achieve effectiveness and safety comparable to those of center-based CR has not been established. Randomized controlled trials with long-term follow-up, including formal economic evaluation, are needed to address these questions.

## Figures and Tables

**Table 1:** Baseline participant characteristics.

Variable	All Participants (*N* = 30)
Male sex	23 (77)
Age, years	61.6 ± 8.9
Body mass index, kg/m^2^	25.4 ± 3.9
Index of cardiovascular disease for inclusion	
Angina pectoris	3 (10)
Chronic heart failure	7 (23)
Myocardial infarction	19 (63)
History of cardiac surgery	1 (3)
Ever-smoker	17 (57)
Comorbidities	
Hypertension	19 (63)
Diabetes mellitus	11 (37)
Dyslipidemia	25 (83)
Medications at enrollment	
Antiplatelet agent	25 (83)
Anticoagulant	2 (7)
β-blocker	27 (90)
ACEi/ARB/ARNI	26 (87)
MRA	8 (27)
SGLT2 inhibitor	7 (23)
Statin	25 (83)
Loop diuretic	6 (20)
Hemodynamics and echocardiography	
Systolic blood pressure, mm Hg	115.6 ± 15.8
Diastolic blood pressure, mm Hg	72.2 ± 9.2
Heart rate, beats/min	72.5 ± 11.6
Left ventricular ejection fraction, %	48 ± 11
Laboratory tests	
Hemoglobin, g/dL	13.1 ± 1.8
Sodium, mEq/L	140.4 ± 1.8
Creatinine, mg/dL	0.89 ± 0.20
N-terminal pro–B-type natriuretic peptide, pg/mL	651 (213, 1147)
Hemoglobin A1c, %	6.1 ± 0.5
Low-density lipoprotein cholesterol, mg/dL	95.1 ± 30.6
Triglyceride, mg/dL	88.3 ± 57.5

Data are presented as mean ± standard deviation for continuous variables, except for N-terminal pro–B-type natriuretic peptide, which is reported as median (interquartile range), and as *n* (%) for categorical variables. ACEi, angiotensin-converting enzyme inhibitor; ARB, angiotensin receptor blocker; ARNI, angiotensin receptor–neprilysin inhibitor; MRA, mineralocorticoid receptor antagonist; SGLT2, sodium–glucose cotransporter 2. One participant with both angina pectoris and chronic heart failure is counted under angina pectoris.

## Data Availability

The data presented in this study are not publicly available. No plan to share individual participant data was specified in the trial registration (jRCTs042230018), and the informed consent obtained from participants did not cover data sharing with third parties. Restrictions also apply under Japan’s Clinical Trials Act and the Act on the Protection of Personal Information.

## Supplementary Materials

The following supporting information can be downloaded at https://www.mdpi.com/article/10.3390/jcm15218658/s1, Table S1: Patient inclusion and exclusion criteria; Table S2: Exercise prescription according to the FITT (frequency, intensity, time, and type) principle; Supplementary Text S1: Additional details on the intervention platform and adherence measurement; Figure S1: Participant flowchart; Supplementary Text S2: Detailed study limitations; Figure S2: Individual adherence to the prescribed exercise sessions; Table S3: Adverse events during the intervention period.

## Abbreviations

The following abbreviations are used in this manuscript:

6MWD	6-min walk distance
CI	Confidence interval
CR	Cardiac rehabilitation
CVD	Cardiovascular disease
HADS	Hospital Anxiety and Depression Scale
IQR	Interquartile range
MCID	Minimal clinically important difference
MET	Metabolic equivalent of task
SD	Standard deviation
VAT	Ventilatory anaerobic threshold
VO_2_	Oxygen uptake
