# Supplementary material for: Feasibility, Safety, Adherence, and Functional Outcomes of a Mobile Application-Facilitated, Unsupervised Remote Cardiac Rehabilitation Program with Exercise Intensity Optimization: A Single-Arm Clinical Trial
Source: J Clin Med. 2026 Nov 6;15(21):8658. doi: 10.3390/jcm15218658 (PMC13763579; doi:10.3390/jcm15218658)
Supplement: Supplementary file 1 [file jcm-15-08658-s001.zip › jcm-4617630-supplementary.pdf]

Supplementary Materials

Table S1. Patient inclusion and exclusion criteria

**Inclusion criteria (all of 1–4 must be met)**
1. Men and women aged ≥18 years
2. Diagnosed with one or more of the following cardiovascular diseases and indicated for cardiac rehabilitation:
(1) acute myocardial infarction or angina pectoris
(2) chronic heart failure
(3) history of cardiac surgery, including coronary artery bypass grafting, valve surgery, and transcatheter aortic valve implantation
(4) arrhythmia or status post implantation of a cardiac implantable electronic device
(5) implantation of an implantable ventricular assist device
(6) history of heart transplantation
(7) pulmonary hypertension
(8) aortic disorder, including aortic dissection, status post aortic surgery, or endovascular aneurysm repair
(9) peripheral artery disease presenting with intermittent claudication
3. Unable to attend, or declined to attend, standard outpatient cardiac rehabilitation
4. Provided written informed consent

Exclusion criteria (any of the following)
1. Unstable angina for which treatment had not been completed
2. Pregnancy or breastfeeding
3. Uncontrolled arrhythmia
4. Symptomatic severe aortic stenosis
5. New York Heart Association functional class IV
6. Pulmonary embolism
7. Myocarditis or pericarditis
8. Acute aortic dissection
9. Living alone
10. Inability to use mobile applications on a smartphone
11. Cardiac rehabilitation not feasible because of difficulty walking or difficulty communicating
12. Respiratory exchange ratio of <1.05 on the baseline cardiopulmonary exercise test

Table S2. Exercise prescription according to the FITT (frequency, intensity, time, and type) principle

Component	Description
Frequency	Three sessions per week for 5 months.

Intensity	Initial level set at the anaerobic threshold derived from a baseline cycle-ergometer cardiopulmonary exercise test (VO_2 converted to METs); eight intensity levels in 0.2-MET increments (mean VO_2 2.4–3.8 METs); target rating of perceived exertion, Borg 11–13.
Time	Approximately 1 h per session (10-min warm-up; main exercise of six sets of 5-min exercise followed by 1-min rest; 10-min cool-down).
Type	Equipment-free, primarily standing aerobic exercises performed at home with video guidance.
Progression	At each rest period, the application adjusted the intensity level by one step when the participant-entered Borg rating fell outside the target range (up when ≤10, down when ≥14); the CR team also adjusted the prescribed level during counseling according to participant progress; participants who reached the highest level (level 8) continued at that level.
Feedback	Post-session summary (resting and end-of-session heart rate, mean heart rate, and mean METs; heart rate from the Apple Watch for reference only) and, for selected menus, a camera-based movement-matching score.
Monitoring	Exercise records and daily blood pressure, pulse rate, body weight, and meal photographs were transmitted to the server and reviewed asynchronously by the CR team on a web-based clinician console; multidisciplinary video counseling every 2 weeks, with additional sessions as needed; in-person visits at baseline and at 1, 3, and 5 months.

CR, cardiac rehabilitation; METs, metabolic equivalents of task; VO_2, oxygen uptake.

Supplementary Text S1. Additional details on the intervention platform and adherence measurement

Heart rate monitoring during exercise

Heart rate during exercise was obtained and displayed using an Apple Watch. Because this device is not a medical device and is susceptible to motion artifacts, the values were used only as reference information for the participant and were not used to adjust exercise intensity. After each session, the application displayed a summary of the exercise record for reference by the participant, including the resting and end-of-session heart rates, mean heart rate during exercise, and mean metabolic equivalents of task performed. As these heart rate values were derived from the Apple Watch, they were provided for reference only.

Movement-matching feedback

For some exercise menus, the application used the smartphone camera to detect the movements of the participant during exercise and provided a score reflecting how closely the performed exercise matched the reference video, as feedback to support correct execution. This score was provided to the participant as feedback only and did not affect the prescribed exercise intensity.

Adherence measurement

An exercise session was counted only when the application logged a completed session; any session without a corresponding application record was treated as not performed. Such missing records were rare because sessions were logged automatically. Adherence was summarized as the total number of application-recorded sessions, as the percentage of prescribed sessions (three per

week over the intervention period), and as the proportion of participants meeting weekly exercise-frequency thresholds (≥1, ≥2, and ≥3 sessions per week). Adherence to other program components, such as daily health-data uploads and counseling sessions, was not formally quantified in this analysis.

Figure S1. Participant flowchart

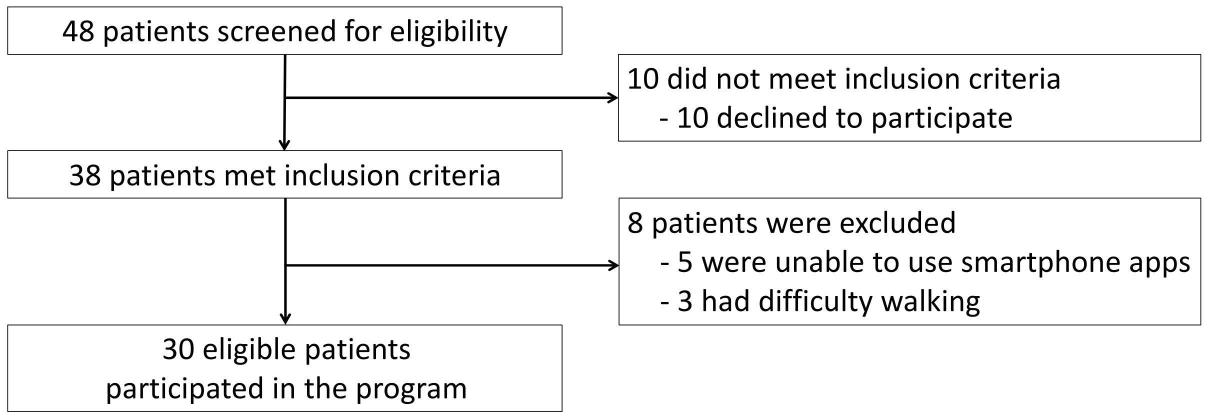

Of the 48 patients assessed for eligibility, 10 did not meet the inclusion criteria because they declined to participate and eight of the remaining 38 patients were excluded (five were unable to use smartphone applications, and three had difficulty walking). The remaining 30 eligible patients participated in the program.

Supplementary Text S2. Detailed study limitations

First, the single-arm design precluded direct comparison with conventional outpatient cardiac rehabilitation (CR) programs, limiting the assessment of relative efficacy. The design also lacked a control group and blinding; therefore, the observed changes cannot be attributed causally to the intervention and may be influenced by the natural course of recovery (particularly after myocardial infarction, the most common index diagnosis), regression to the mean, or a learning effect on the 6-min walk distance. Randomized controlled trials comparing remote and conventional CR modalities are, therefore, warranted to validate these findings.

Second, although participants completed a median of 89.5% of the prescribed sessions during the 5-month intervention period, the long-term sustainability of participation, as well as its benefits on cardiovascular outcomes and mortality, remains unclear. Further studies are needed to determine whether participants maintain exercise habits and continue to derive benefits beyond the study period.

Third, when the anaerobic threshold level prescribed from cycle-ergometer cardiopulmonary exercise testing is applied directly to standing exercise, the initial exercise intensity may be underestimated because the metabolic cost of weight-bearing upright exercise is relatively higher. Optimization of prescription algorithms that account for the difference in metabolic equivalents of task between exercise modalities is a subject for future work.

Fourth, the wearable device used for heart rate monitoring during exercise (Apple Watch) is not a medical device and the heart rate obtained could serve only as reference information. More reliable verification of exercise load and management of exercise intensity will require a medical device that can display and record heart rate.

Fifth, ensuring safety is the most important issue in remote CR. Unlike supervised center-based CR, a remote, home-based setting makes it difficult to respond on site to acute events such as arrhythmia, falls, syncope, and cardiac arrest and real-time coordination with medical professionals and institutions is also limited. The present study did not establish a system for responding to such acute events. Building a real-time monitoring system during exercise and establishing procedures for promptly contacting caregivers (such as family members) and emergency medical services are considered essential for the safe implementation of remote CR in the future.

Sixth, adherence was quantified only for the exercise-session component. Adherence was summarized cumulatively over the 5-month intervention period, and temporal trends in adherence were not analyzed; application usability was not assessed with a validated instrument. Engagement with the other components of this multicomponent program—daily uploads of blood pressure, pulse rate, and body weight; meal photographs; attendance at multidisciplinary counseling every 2 weeks; and clinician review and adjustment of the exercise prescription—was not systematically measured. Consequently, the available evidence cannot determine to what extent the observed improvements reflect sustained engagement with the full program rather than the exercise component alone, and the overall feasibility of the multicomponent intervention could not be fully characterized. Future studies should prospectively capture component-level engagement metrics to clarify which elements drive the observed benefits. In addition, although basic psychosocial support, including encouragement and supportive listening, was provided during multidisciplinary counseling, no structured, protocol-defined psychological or social-support intervention was included in the program. Pharmacological therapy was managed by the participants' attending physicians as part of routine outpatient care, without a study-specific optimization protocol;

although initiations and discontinuations of concomitant medications were recorded on the case report forms, dose adjustments were not systematically collected in the case report forms, and the influence of medication changes on the outcomes was not analyzed. Guidance on medication adherence was provided during outpatient visits and multidisciplinary counseling sessions, but its content and frequency were not systematically recorded for research purposes; therefore, the fidelity of these non-exercise components could not be assessed, and the observed changes cannot be attributed to the exercise intervention alone.

Seventh, the study was conducted at a single center with a small sample, smaller than originally planned, limiting the precision and generalizability of the findings. Because enrollment was restricted to patients able and willing to use a smartphone application, selection bias may limit the applicability of the findings to the broader population of patients with cardiovascular disease. In addition, the cohort was clinically heterogeneous, including patients with myocardial infarction, chronic heart failure, angina pectoris, and a history of cardiac surgery, and the interval from the index hospitalization to enrollment was not systematically recorded; diagnosis-specific analyses were not feasible given the sample size. No participant was enrolled with an arrhythmia, such as atrial fibrillation, as the index condition.

Eighth, because this study was funded by CaTe Inc., the developer of the evaluated program, and several authors are affiliated with the company, the potential for sponsor-related bias should be considered when interpreting the results. To minimize this risk, the protocol, including the prespecified primary endpoint, was approved by the Certified Review Board, which also reviewed the conflict-of-interest management plan under the Clinical Trials Act, and the study was registered before enrollment. Outcome measurements, including the 6-min walk test and cardiopulmonary exercise testing, were performed by clinical staff at Fujita Health University

Hospital. Data management and monitoring, including verification of case report form data against medical records, were performed by an external contract research organization, and an external audit was conducted. The statistical analysis for the study report was requested from the designated analysis manager at Fujita Health University after database lock.

Finally, the small sample size was insufficient to establish the safety of the program, and safety events were evaluated by the attending physician, who also conducted the study visits, rather than by an independent committee. Therefore, these findings should be considered preliminary and require confirmation in larger, controlled, multicenter studies.

Figure S2. Individual adherence to the prescribed exercise sessions

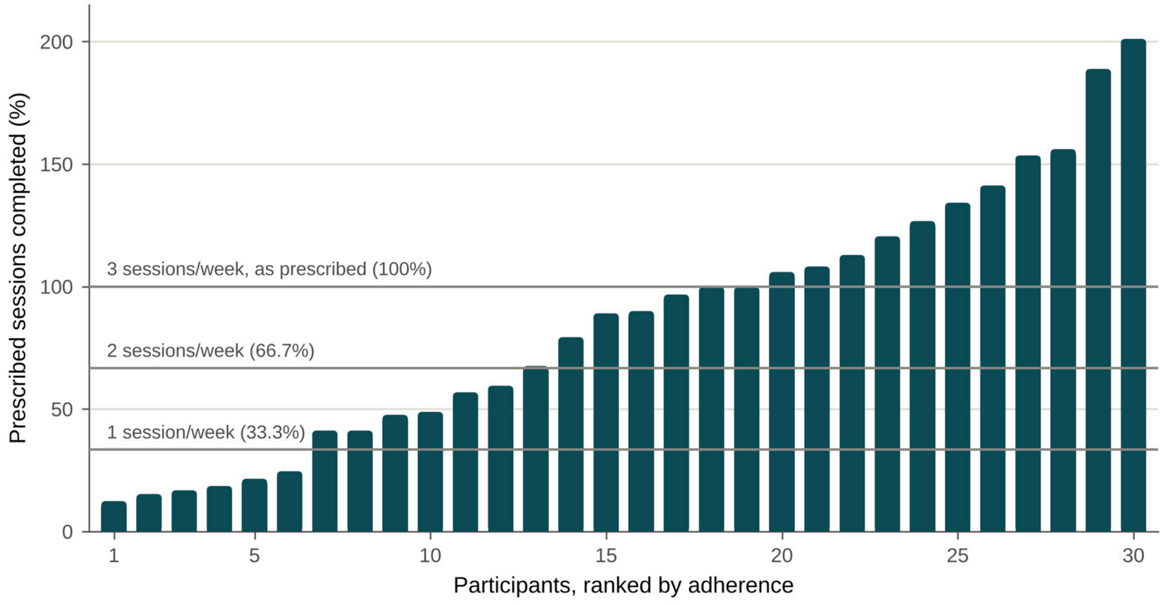

Each bar represents one participant: the number of application-recorded exercise sessions as a percentage of the prescribed sessions (three per week over that participant's intervention period). Participants are ranked in ascending order. Horizontal lines indicate 33.3%, 66.7%, and 100% of the prescribed sessions, corresponding to an average of one, two, and three sessions per week, respectively. The median was 89.5% (interquartile range, 42.8–118.6%; range, 12.4–201.1%).

Table S3. Adverse events during the intervention period

Participant	Adverse event	Day of onset	Severity	Relationship to the program
1	Sleep apnea syndrome	133	Mild	Unrelated
2	Dizziness	135	Mild	Unrelated
3	Dizziness	27	Mild	Unrelated
3	Chest pain	139	Mild	Unrelated
4	Nausea	58	Mild	Unrelated
5	Cough	111	Mild	Unrelated
5	Hematochezia	111	Mild	Unrelated
6	Dizziness	20	Mild	Unrelated
7	Chest tightness	12	Mild	Unrelated
7	Exertional dyspnea	54	Mild	Unrelated
8	Exertional dyspnea	17	Mild	Unrelated
9	Chest pressure	20	Mild	Unrelated
9	Hematuria	69	Mild	Unrelated
10	Chest discomfort	17	Mild	Unrelated
10	Palpitations	17	Mild	Unrelated
10	Stomach pain	101	Mild	Unrelated
11	Pruritus of a rash on the foot	89	Mild	Unrelated
12	Fatigue	130	Mild	Unrelated
12	Hyposmia	130	Mild	Unrelated
12	SARS-CoV-2 pneumonia	130	Moderate	Unrelated
13	Constipation	24	Mild	Unrelated
13	Cough	80	Mild	Unrelated
14	Abnormal chest sensation	0	Mild	Unrelated
14	Bilateral finger numbness	65	Mild	Unrelated
14	Bilateral toe numbness	65	Mild	Unrelated
15	Dyspnea	30	Mild	Unrelated
16	Exertional dyspnea	47	Mild	Unrelated
16	Pleural effusion	48	Mild	Unrelated
16	Worsening heart failure[a]	57	Moderate	Unrelated
16	Right nipple pain	85	Mild	Unrelated
16	Chest pain	109	Mild	Unrelated
16	Lower-extremity edema	109	Mild	Unrelated
16	Shivering	145	Mild	Unrelated
17	Dysgeusia	12	Mild	Unrelated

18	Abnormal chest sensation	12	Mild	Unrelated
18	Chills	12	Mild	Unrelated
18	Nausea	12	Mild	Unrelated

Adverse events with onset from the start of the program to the end of the 5-month intervention period are listed (37 events in 18 participants). Day of onset is counted from the start of the program (day 0). Severity was graded as specified in the protocol: mild, not interfering with daily life; moderate, interfering with daily life to some extent; and severe, preventing daily life. The causal relationship to the program was judged by the investigator as related or unrelated. None of the events led to discontinuation of the program. [a] Serious adverse event (hospitalization); see Section 3.5 of the main text. Four additional events with onset before the start of the program (right shoulder pain, COVID-19, left pyelonephritis, and nocturnal cough; all mild and unrelated) are not listed.
